# Targeting Cholesterol Homeostasis Improves Recovery in Experimental Optic Neuritis

**DOI:** 10.3390/biom12101437

**Published:** 2022-10-07

**Authors:** Cheyanne R. Godwin, Jeffrey J. Anders, Lin Cheng, Benjamin W. Elwood, Randy H. Kardon, Oliver W. Gramlich

**Affiliations:** 1 Department of Ophthalmology and Visual Sciences, University of Iowa, Iowa City, IA 52242, USA; 2 Center for the Prevention and Treatment of Visual Loss, Iowa City VA Health Care System, Iowa City, IA 52246, USA; 3 Department of Neuroscience and Pharmacology, University of Iowa, Iowa City, IA 52242, USA

**Keywords:** optic neuritis, multiple sclerosis, gentisic acid, EAE, electrophysiology, cholesterol recycling, RGC, organoids

## Abstract

Acute optic neuritis (ON) is a common cause of vision loss and is often associated with multiple sclerosis (MS). Cholesterol recycling has been identified as a key limiting factor in recovery after demyelination events. Thus, the purpose of our study was to determine if the augmentation of cholesterol transport by gentisic acid (GA) benefits retinal ganglion cell (RGC) development and myelination in organoid systems and enables the recovery of the ocular phenotype upon systemic GA treatment in a MOG-induced experimental autoimmune encephalomyelitis (EAE) ON model. The retinal organoids treated with GA demonstrate an accelerated maturation when compared to the conventionally derived organoids, which was evidenced by the improved organization of Brn3a-GFP^+^RGC and increased synaptogenesis. A GA supplementation in brain organoids leads to a 10-fold increase in NG2 and Olig2 expression. Weekly GA injections of EAE mice significantly lessened motor-sensory impairment, protected amplitudes in pattern electroretinogram recordings, and preserved visual acuity over the study period of 56 days. Furthermore, GA-treated EAE mice revealed diminished GCL/IPL complex thinning when compared to the untreated EAE mice. An optic nerve histopathology revealed less severe grades of demyelination in the GA-treated EAE cohort and fewer infiltrating cells were observed. Interventions to improve cholesterol homeostasis may be a viable approach to promoting the rehabilitation of MS patients.

## 1. Introduction

Acute optic neuritis (ON) is a common cause of vision loss in autoimmune-mediated demyelination disorders and is frequently associated with multiple sclerosis (MS) [1]. ON is the initial presenting symptom in approx. 25% of new MS cases, and 70% of MS patients experience at least one episode of ON during disease progression, of which the risk of recurring ON is 35% [2,3,4]. ON most characteristically presents with acute, painful, and unilateral vision loss [5]. In MS-associated ON, vision loss is often moderate with better than 20/200 vision and with a recovery up to 20/40 or better in 95% of patients [1,5]. However, even while the recovery of vision is generally favorable, MS patients frequently report deficits in other aspects of vision such as color vision, contrast detection, and the visual field [3,6]. Moreover, the recovery of vision in MS cases that present with recurring or bilateral ON is often delayed and limited. More importantly, even MS patients with no history of ON show evidence of retinal ganglion cell (RGC) loss characterized by the thinning of the retinal nerve fiber layer and RGG layer as measured by optical coherence tomography (OCT) imaging [7,8].

In MS, the inflammation of the optic nerve leads to demyelination and vision loss; this inflammation is thought to be primarily T cell-mediated and thus current therapies are aimed at immunomodulation [9,10]. These treatments include high-dose corticosteroids for acute attacks, and disease-modifying therapies (DMTs) for chronic disease and treatment escalation [9,11,12]. DMTs have been shown to reduce disease progression and relapses in MS by reducing the number of T cells, the degree of T cell activation, and the level of cytokine production [13]. As with MS, the current treatment for acute ON is high-dose corticosteroids, which have been shown to improve the time, but not the degree, of recovery [1,3,5]. Intravenous immunoglobulin injections have been attempted in cases of MS-ON refectory to steroid therapy; however, studies have shown mixed results, with one study demonstrating no improvement in visual function [1] and another showing a return to normal visual function after 5 months of treatment [14]. Plasma exchange therapy has also been attempted in a limited study of patients with ON that were refractory to steroid therapy with some apparent short-term improvement in visual acuity, but this effect was not sustained in all patients [15]. Another treatment avenue currently in clinical trials is mesenchymal or hematopoietic stem cell-based therapy [16], or anti-CD20 antibody therapy, with the intent to reduce the severity of autoimmunity (clinicaltrials.gov). Three major anti-CD20 drugs, namely, Rituximab, Ocrelizumab, and Ofatumumab, have been approved for the treatment of MS [17].

Research groups recently identified alterations in cholesterol homeostasis and transport in MOG-induced Experimental Autoimmune Encephalomyelitis (EAE)-ON [18,19] and revealed that systemic MSC therapy in EAE augmented these pathways resulting in the amelioration of vision loss and motor-sensory impairment [20]. These in vivo studies have suggested that the clearance of cholesterol-rich myelin debris by microglia is essential for reducing the inflammation of the CNS after demyelinating events [19,21]. While cholesterol recycling and transport after demyelination are the limiting factors in myelin repair, maintaining cholesterol homeostasis is also vital for preserving retinal function and structure [22,23]. As these studies indicate, cholesterol is locally synthesized within the CNS and is essential for synaptogenesis, particularly in RGC, synaptic plasticity, and normal retinal structure and function including presynaptic neuronal differentiation. Thus, the disruption of cholesterol homeostasis and transport contributes significantly to retinal degeneration and visual impairment [22,23,24,25].

After demyelinating events, microglia phagocytize the cholesterol-rich myelin debris, digest it, and export the cholesterol to the extracellular space [21]. This process is accomplished via the efflux transporter ATP-binding cassette A1 (Abca1), which is a key cholesterol transporter and is likely important in modulating cholesterol homeostasis [25]. Studies show that the knock-out of *Abca1* in the retinas of mice leads to retinal degeneration [25,26]. However, during optic nerve demyelination, the accumulation of intracellular cholesterol could exceed the efflux transporter’s capacity, causing the microglia to become lipid-laden and develop into proinflammatory foam cells [21]. The impaired recycling of cholesterol via *Abca1* significantly dampens myelin sheath repair and *Abca1* expression has been found to be significantly downregulated in EAE-ON mouse models [20,21].

Cholesterol recycling, particularly via Abca1, is required to maintain retinal function and the disruption of this pathway leads to retinal degeneration. In a study of age-related macular degeneration, researchers found that Abca1 expression can be increased by the systemic application of gentisic acid (GA; 2,5 dihydroxybenzoic acid) [27]. GA is an aspirin derivative with anti-inflammatory, antigenotoxic, antioxidant, antimicrobial, and neuroprotective activities [27,28]. Furthermore, GA, similar to salicylic acid, can inhibit prostaglandin synthesis [29] as one of the key mechanisms of non-steroidal anti-inflammatory drugs (see Thieme’s chemical encyclopedia at roempp.thieme.de).

An alteration in *Abca1* expression is seen in EAE-ON [20]; thus, the purpose of our study was to determine if the augmentation of Abca1 by GA influences myelination and the recovery of the ocular phenotype in EAE-ON. Since GA has been shown to upregulate Abca1 expression, we first used GA to increase Abca1 expression in vitro using induced pluripotent stem cell (iPSC)-derived retinas and brain organoid systems to determine the effects on cholesterol synthesis and transport, neurogenesis, synaptogenesis, and myelination. We found that the administration of GA in organ-specific organoids accelerated RGC development, synaptogenesis, cholesterol synthesis, and myelination. We then proceeded to evaluate the effects of a weekly systemic GA administration in our MOG-induced EAE-ON model to ameliorate the severity of the disease progression and on functional and structural recovery in the visual system. We determined that the GA administration lessened the motor-sensory and visual impairment in EAE animals, which was accompanied by increased *Abca1* expression and reduced optic nerve demyelination.

Our in vitro and in vivo results suggest that the augmentation of cholesterol homoeostasis and transport via GA-mediated Abca1 expression offers a promising new therapeutic avenue for MS.

## 2. Materials and Methods

### 2.1. Human iPSC-Derived Retinal and Brain Organoids

**Figure 1:**
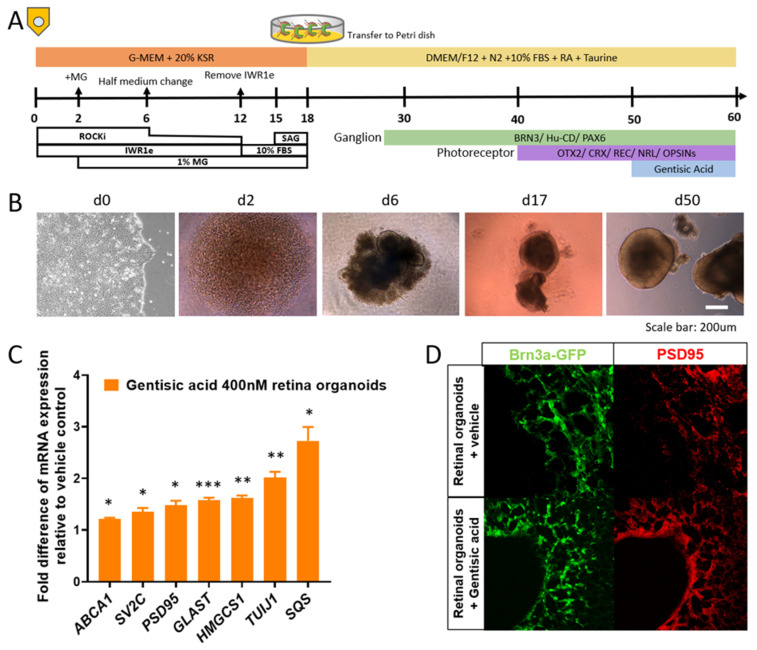
Administration of gentisic acid (GA) increases cholesterol synthesis and accelerates RGC maturation and synaptic plasticity in retinal organoids: (**A**) Retinal organoid production and treatment timeline with GA application on days 50–60; (**B**) Progression of growth from iPSC to retinal organoids; (**C**) RT-PCR analysis reveals significantly increased expression of cholesterol synthesis-related markers (*HMGCS1, SQS*), synaptic plasticity-related proteins (*SV2C, PSD95, GLAST,* and *TUJ1*), and *ABCA1* in GA-treated retinal organoids when compared to conventionally grown retinal organoids. * *p* < 0.05, ** *p* < 0.01, and *** *p* < 0.001 (**D**) Representative, confirmatory immunofluorescence images show increased expression of a synaptic plasticity-related marker (PSD95) and a more matured organization of Brn3a-GFP^+^RGC after GA supplementation.

Retinal organoids were derived from human iPSC [30]. Human iPSCs were induced to express Brn3a^-^GFP via CRISPER/Cas9 using a previously described protocol [31] prior to organoid induction. Starting at day 0, iPSCs were detached from Matrigel-coated plate with mTeSR plus (Stem Cell Technologies, Vancouver, BC, Canada) using TrypLE Express (Invitrogen, Carlsbad, CA, USA) containing 0.05 mg/mL DNase I (Roche, Basel, Switzerland) and 10 μM Y-27632 (Sigma, St. Louis, MO, USA, SML1045-5MG). Cells were quickly reaggregated using low-cell-adhesion 96-well plates with V-bottomed conical wells in retinal differentiation medium (12,000 cells/well, 100 μL) containing 20 μM Y-27632. The retinal differentiation medium was G-MEM (Thermo Fisher, Waltham, MA, USA) supplemented with 20% Knockout Serum Replacement (Thermo Fisher), 0.1 mM of nonessential amino acids (gibco), 1 mM of pyruvate (gibco), 0.1 mM of 2-mercaptoethanol (gibco), and 100 U/mL of penicillin-streptomycin (gibco). IWR1e (Merck, Darmstadt, Germany) was added from day 0 to day 12 for a final concentration of 3 μM. Matrigel (growth-factor-reduced; final 1% *v*/*v*; BD Biosciences, Franklin Lakes, NJ, USA) was added from day 2 to day 18. On culture day 12, floating aggregates were transferred to a floating petri dish. FBS (final 10% *v*/*v*) was added to medium from day 12 to day 18. SAG (100 nM, Enzo Life Sciences, Farmingdale, NY, USA) was added to medium from day 15 to day 18. On day 18, the culture was transferred to an NR culture medium (DMEM/F12-Glutamax medium (gibco) containing the N2 supplement (Thermo Fisher) and 100 U/mL penicillin-streptomycin) for long term culture. On day 50, retinal organoids were transferred to 48-well plates. On days 50–60, organoids were cultured with 1 mL NR medium/organoid supplemented with GA (Sigma, 149357) at a final concentration of 400 nM, as well as vehicle control. The NR medium was changed every 2–3 days. On day 60, retinal organoids were harvested for RT-PCR (8 organoids) and immunofluorescence (3–4 organoids; Figure 1a,b).

**Figure 2:**
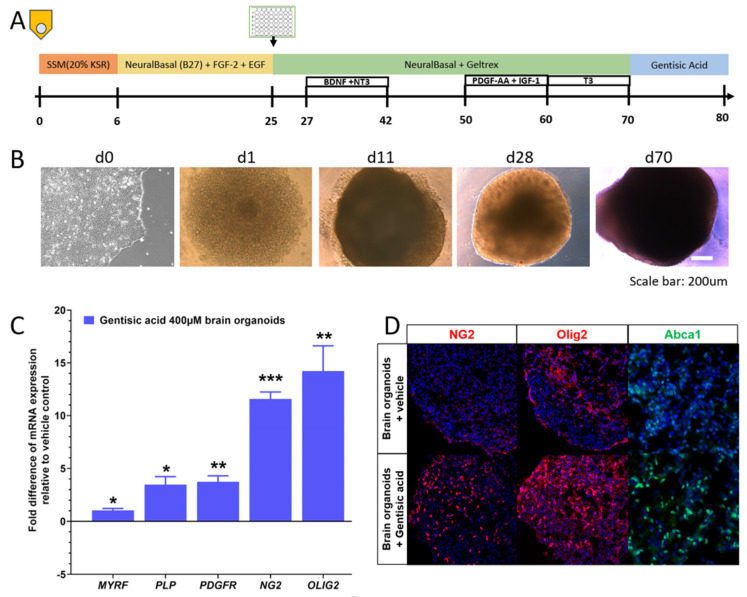
Gentisic acid (GA) supplementation accelerates myelination in brain organoids: (**A**) Schematic representation of the timeline of brain organoid generation, including GA application at days 70–80. (**B**) Representative brightfield images document progression of organoid growth from iPSC to brain organoids. (**C**) RT-PCR analysis demonstrates significantly increased expression of oligodendrocyte (precursor) cell markers (*NG2, OLIG2*, and *PDGFR*) and genes related to myelin formation (*MYRF* and *PLP*) after GA supplementation when compared to conventionally generated brain organoids. * *p* < 0.05, ** *p* < 0.01, and *** *p* < 0.001 (**D**) Representative images display increased numbers of NG2^+^ and Olig2^+^ oligodendrocytes and increased expression of *ABCA1* in GA supplemented brain organoids.

Brain organoids were derived from human iPSCs using a previously described method [32]. Briefly, intact colonies were transferred to low adherence 96-well plates with spheroid starter media, 10 μM of Y-27632, 10 μM of dorsomorphin (Sigma, P5499-5G), and 10 μM of SB-431542 (Sigma, s4317-5MG). Media were changed to Neurobasal-A (gibco) media with FGF-2 (R&D Systems, Minneapolis, MN, USA) and EGF (R&D Systems) from day 6 to day 25. On day 25, spheroids were transferred to ultra-low attachment 6-well plates with Geltrex. BDNF (R&D Systems) and NT-3 (R&D Systems) were added from day 27 to day 42. At day 50, PDGF-AA (R&D Systems) and IGF-1 (R&D Systems) were added. At day 60, T3 (Sigma) was added. Around day 70, the spheroids were mature with sufficient yield of oligodendrocyte precursor cells and oligodendrocytes and were maintained in Neurobasal-A spheroid media. On days 70–80, GA at a final concentration of 400 μM and vehicle control were applied to Neurobasal-A spheroid media plus 1% Geltrex (Invitrogen). On day 80, oligocortical spheroids were harvested for RT-PCR and immunofluorescence (Figure 2a,b).

### 2.2. RT-PCR

RNA was isolated from organoids using RNeasy columns (QIAGEN, Hilden, Germany). Isolated RNA was converted to cDNA using SuperScript Reserve Transcriptase (Thermo Fisher) according to the manufacturer’s instructions. Quantitative polymerase chain reaction (qPCR) was performed with SsoFast™ EvaGreen^®^ Supermix (BioRad, Hercules, CA, USA) through a CFX96 204 Real-Time PCR System (Bio-Rad) with specific primers listed in Table 1. Amplification was normalized against that of the Tata Box-binding protein (TBP). Expression changes were calculated using the ddCT method [33].

### 2.3. Organoid Immunohistochemistry

Organoids were fixed in 4% PFA for 30 min, infiltrated with sucrose, and embedded in Optimal Cutting-Temperature (OCT) media (Fisher Scientific, Waltham, MA, USA) for cryo-sectioning. Seven-micron thick sections were taken and blocked in 1%BSA/PBS-0.3% Triton X100 for 1 h at room temperature. Organoid sections were incubated either with PSD95 (ab12093, abcam, Waltham, MA, USA), NG2 (ab275024, abcam), Olig2 (ab136253, abcam), or Abca1 (PA1 16789, Thermo Fisher) antibodies diluted 1:250 in blocking solution at 4 °C overnight. Slides were washed and incubated for three hours at room temperature in appropriate secondary antibody (A11055, Invitrogen; ab6718, abcam) diluted 1:500 in blocking solution. Slides were washed twice for 5 min in PBS, incubated for 5 min in 1:1000 DAPI, washed twice more with PBS, and cover slipped. Slides were imaged using a confocal microscope (Zeiss LSM 880 confocal microscope, Carl Zeiss AG, Oberkochen, Germany).

### 2.4. Animals and Study Design

**Figure 3:**
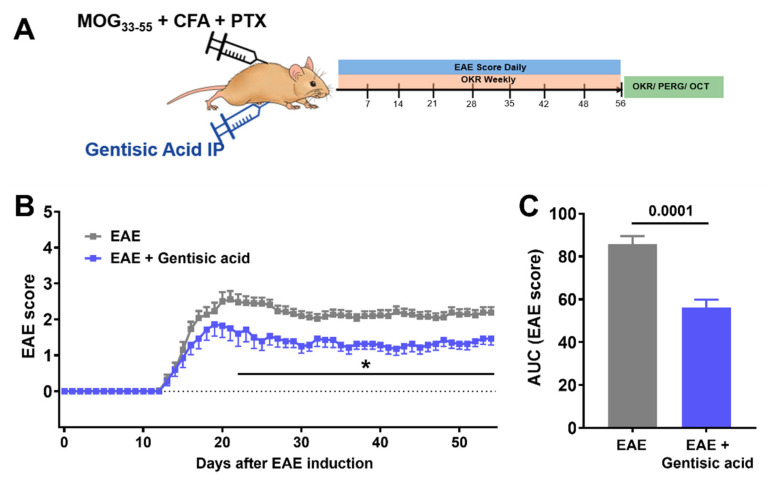
Systemic gentisic acid (GA) administration reduces clinical severity in MOG-induced EAE: (**A**) Overview of animal study design including EAE induction in 52 female C57BL6/J mice and in vivo readouts. Twenty-six EAE mice received weekly intraperitoneal (ip) administration of 50 mg/kg GA. All animals underwent daily EAE scoring, weekly assessment of visual acuity, and pattern ERG recordings and OCT imaging 56 days after EAE induction. (**B**) Untreated EAE mice (grey = EAE) displayed a moderate course of motor sensory impairment over time. Systemic GA administration in EAE animals (blue = EAE + GA) mitigated motor-sensory deficits and resulted in a significantly milder disease progression. Mean scores per day with SEM are given and * indicates *p* < 0.05 in EAE vs. EAE + gentisic acid treatments. (**C**) Analysis of area under curve (AUC), as the sum of individual EAE courses per group, confirmed a significantly milder overall disease progression in GA-treated EAE mice when compared to untreated, vehicle-administered EAE animals.

All animal experiments were approved by the Iowa City VA IACUC and were conducted according to the ARVO Statement for the Use of Animals in Ophthalmic and Vision Research. Female C57BL6/J (B6) mice were housed at the Iowa City VAMC animal facility with a 12 h light–dark cycle and food ad libitum. EAE-ON was induced in 52 female mice using MOG immunization in two subsequent cohorts (15 EAE mice in cohort 1 and 15 EAE mice in cohort 2). A total of twenty-three EAE mice received 50 mg/kg of GA intraperitoneally on day 0 weekly (EAE + GA: 8 GA-treated EAE mice in cohort 1 and 15 GA-treated EAE mice in cohort 2) and then once per week for 8 weeks according to previously published work on GA pharmacokinetics [27,34]. Untreated EAE mice received an equivalent volume of vehicle solution. Another 20 naïve, age-matched mice served as controls. Disease course progression was measured daily using a 5-point scoring system. Visual acuity was measured weekly using optokinetic response (OKR). Fifty-six days after EAE induction, all animals underwent pattern electroretinogram (pattern ERG) recordings and ocular coherence tomography (OCT) imaging prior to euthanasia (Figure 3a). Assessment of outcome parameters was performed by trained personnel who were blinded to the study groups (EAE and naïve) and treatment assignment (placebo vs. GA treatment) to minimize potential bias. All animals were euthanized by CO_2_ inhalation followed by cervical dislocation.

### 2.5. EAE-ON Model

EAE-ON was induced in eight to twelve-week-old female C57BL6/J (B6) mice using a previously described method [20,35]. Briefly, mice were injected subcutaneously at two sites along the back with 200 μg of MOG_35–55_ (Sigma Aldrich, St. Louis, MO, USA) emulsified with complete Freund’s adjuvant (Sigma Aldrich) containing 2 mg/mL of mycobacterium tuberculosis (BD Difco, Franklin Lakes, NJ, USA). Animals were injected intraperitoneally with 200 ng of pertussis toxin (Sigma Aldrich) at day 0 and day 2. Motor-sensory function was assessed daily by a trained investigator who was masked to the study groups and treatment assignment using a 5-point scoring system with the following criteria: 0 = no symptoms, 0.5 = partial tail paralysis, 1 = tail paralysis, 1.5 = partial tail paralysis and waddling gait, 2 = tail paralysis and waddling gait, 2.5 = partial limb paralysis, 3 = paralysis of one limb, 3.5 = paralysis of one limb and partial paralysis of another, 4 = paralysis of two limbs, 4.5 = moribund state, and 5 = death.

### 2.6. Optokinetic Tracking Response (OKR)

Bilateral visual acuity was measured in subsets of 14 EAE mice, 15 GA-treated EAE mice, and 10 naive controls using the OptoDrum system (Striatech, Tübingen, Germany) [36,37]. Awake mice were placed on a platform in a closed chamber with a virtual cylinder projecting stripes of varying spatial frequencies at 99.8% contrast. Mice were observed through a top-down camera and reflexive head movements were detected by automated software. Since rodents track objects in one direction with each eye, eyes were tested individually by alternating the direction of rotation of the cylinder. Lack of reflexive head movements indicates the pattern is no longer being perceived and is used to delineate the threshold of visual ability for the measured eye. The highest spatial frequency as cycles/degree (c/d) detected by each eye reflected visual function.

### 2.7. Pattern Electroretinogram (Pattern ERG)

At week 8, mice of EAE cohort 1 (14 EAE, 7 EAE + GA, and 10 naïve controls) were anesthetized with intraperitoneal injection of ketamine (Mylan, Canonsburg, PA, USA), xylazine (Akorn Inc., Lake Forest, IL, USA) and acepromazine (Rattlesnake Drugs, Scottsdale, AZ, USA). Pupils were dilated using 1% tropicamide and GenTeal gel (Alcon Laboratories, Fort Worth, TX, USA) was placed on the corneal surface for preservation of corneal integrity. Animals were kept on heating pads to maintain constant body temperature between 37 and 38 °C. Pattern ERG was evoked by presenting alternating black and white vertical stimuli at 1 Hz to each eye using a JORVEC (Intelligent Hearing Systems, Miami, FL, USA) system, as previously described [20,38]. Three hundred seventy-two averaged signals with cutoff filter frequencies of 1 to 300 Hz were recorded under mesopic conditions without dark adaptation. Amplitudes (μV) were calculated from the P1 peak to the N2 trough as a representation of RGC electrical function.

### 2.8. Optical Coherence Tomography (OCT)

OCT was used to analyze the structure of the retina in vivo and the measurement of the ganglion cell layer, including the retinal never fiber layer (RNFL) and the inner plexiform layer (GCL/IPL) complex; thickness was measured as described previously [20]. Briefly, borders of the RNFL and the IPL were determined manually, and thickness was measured at a distance of 400 µm away from the center of the optic nerve head in the superior, inferior, nasal, and temporal quadrants. At week 8, all remaining mice of both cohorts were anesthetized with intraperitoneal injection of ketamine (30 mg/kg, Mylan) with xylazine (5 mg/kg, Akorn Inc.). Pupils were dilated using 1% tropicamide (Alcon). OCT images were obtained using an SD-OCT instrument (Bioptigen, Morrisville, NC, USA) and the average regional GCL/IPL complex thickness was calculated as an indicator of RGC degeneration. Data of mouse eyes from 46 naïve animals, 49 EAE mice, and 28 EAE mice that underwent GA treatment were used for statistics. OCT data were excluded either because of poor images or questionable detection of borders between retinal layers.

### 2.9. Optic Nerve Histopathology

Optic nerves and spinal cord tissue were harvested from all mice at the end of the study. Optic nerves were fixed in paraformaldehyde and embedded in paraffin. Optic nerves were sectioned longitudinally at 7 microns. Three to five sections from each optic nerve were then stained with Luxol Fast Blue (LFB); the degree of demyelination was graded on a 3-point scale with 0 representing full myelination and 3 representing complete demyelination. Similar amounts of sections were stained with LFB/H&E to examine potential cellular infiltration and quantity of foam cells. Cellular infiltration was graded on a 4-point scale where 0 indicated no cellular infiltration. Foam cells were identified by their classic morphology with a bubbly cytoplasm from ingested lipid materials [39]. Remaining optic nerve tissue was used for immunofluorescent staining of Abca1^+^ cells.

### 2.10. Statistics

RT-PCR data were analyzed using 1-way ANOVA followed by a Tukey post hoc test. All in vivo data were assessed by trained investigators who were masked to animal groups and treatment regimes. Data were tested for standard distribution and EAE scores were analyzed using a 2-way ANOVA and Tukey post hoc test. Overall disease severity for EAE animals was calculated using area under the curve (AUC) and differences between GA-treated and untreated EAE mice were determined using Student’s t-test. OKR data were analyzed with a 2-way ANOVA followed by a post hoc test for multiple measurements. PERG amplitudes and GCL/IPL complex thickness were analyzed using a 1-way ANOVA followed by a Tukey post hoc test.

Ordinal data for grading of demyelination and cellular infiltration were analyzed using Kruskal–Wallis test. Numeric histopathology data were analyzed using 1-way ANOVA followed by a Tukey post hoc test. Results are given as mean ± standard deviation (SD) except for graphs of longitudinal EAE scoring and visual acuity data for which the standard error of the mean (SEM) is presented. Calculations were performed using GraphPad Prism and *p* values < 0.05 were considered statistically significant.

## 3. Results

### 3.1. Gentisic Acid Accelerates Retinal Cup Maturation

Following the treatment with GA for 10 days, the retinal organoids demonstrated accelerated maturation with increased cholesterol synthesis and synapse formation. The gene expression for markers related to cholesterol synthesis and synaptic markers was quantified via RT-PCR (Table 1). The organoids treated with GA had an increased expression of 3-hydroxy-3-methylglutaryl coenzyme A synthase 1 (*HMGCS1*) and Squalene synthase (*SQS*) relative to the vehicle-treated, conventionally originated organoids. The GA-treated organoids also demonstrated an increased expression of synaptic markers such as Postsynaptic density protein 95 (*PSD95*), Synaptic vesicle glycoprotein 2 (*SV2C*), Glutamine Aspartate Transporter (*GLAST*), and the neuronal marker Beta-Tubulin III (*TUJ1*) (Figure 1c). The organoids expressed GFP under the Brn3a promotor and were examined with immunofluorescence for RGC development as well as synapse formation using PSD95. The treated organoids were found to have an increased expression of PSD95 as well as a more matured organization of Brn3a-GFP^+^ cells compared to the untreated organoids (Figure 1d).

### 3.2. Gentisic Acid Increases Myelination in Brain Organoids

To gain insight into the effects GA has on myelination, brain organoids were grown from human iPSCs using an established model, with GA added to half of these at day 70 for 10 days, and then analyzed with RT-PCR and immunofluorescence. The organoids treated with GA demonstrated a greater than 10-fold increase in oligodendrocyte markers Neuron-glial antigen 2 (*NG2*), and Oligodendrocyte transcription factor 2 (*OLIG2*) expression. The organoids also demonstrated an increase in platelet-derived growth factor receptor (*PDGFR*), an oligodendrocyte precursor cell marker, after the GA treatment. The treated organoids showed an increased expression of myelin-associated markers such as the proteolipid protein (*PLP*) and the myelin regulation factor (*MYRF)* compared to the conventionally grown organoids (Figure 2c). An increase in myelination with the GA treatment was confirmed in the organoids using immunofluorescence for NG2 and Olig2, which revealed an increased expression. The organoids also showed increased Abca1 expression when treated with GA (Figure 2d).

### 3.3. Systemic Gentisic Acid Administration Mitigates Motor-Sensory Deficits in EAE Mice

After observing increased levels of myelination, Abca1 expression, and retinal maturation with GA administered in vitro, we wanted to further investigate GA’s affects in vivo and its potential to reduce motor sensory impairments in MS-like ON. Following EAE induction using MOG_35–55_ immunization, all the mice showed a monophasic disease course with the first clinical signs present at approximately day 12 and a peak motor-sensory impairment at approximately day 20 (Figure 3b). The EAE animals treated with GA still systemically demonstrated motor-sensory impairments with an onset around day 12 and a peak at approximately day 19. Directly after peak disability, however, the GA-treated animals began displaying improvements in their motor-sensory functions, which were then maintained for the remainder of the study (Figure 3b). Throughout a time course of 56 days after the EAE induction, the GA-treated animals showed a reduced overall progression when compared to the untreated EAE mice (area under curve EAE: 86 ± 4 vs. EAE + GA: 56 ± 4, *p* = 0.0001; Figure 3c). Of note, 5 of 23 EAE mice in the GA-treated cohorts deceased compared to 2 out of the 29 untreated, vehicle-injected EAE animals.

### 3.4. Gentisic Acid Treatment Improves Visual Structure and Function in EAE Mice

**Figure 4:**
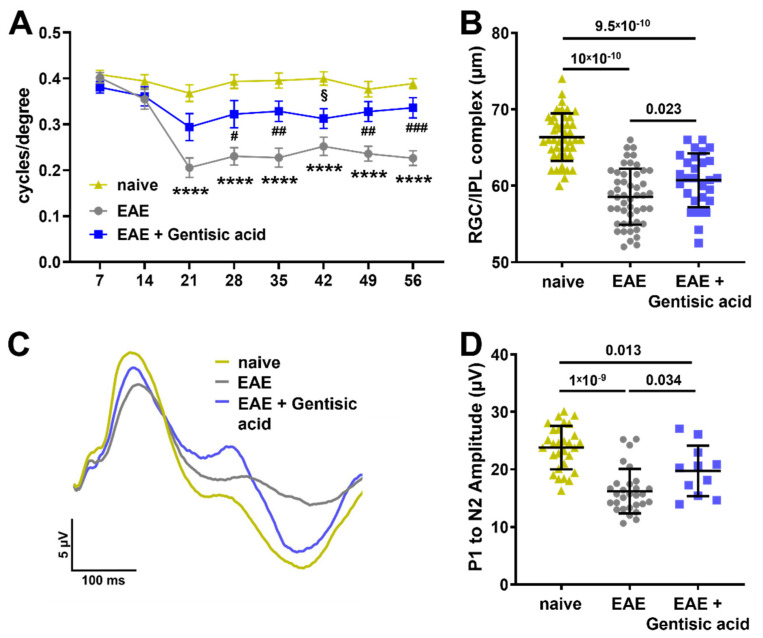
Gentisic acid (GA) treatment in EAE animals improves visual function and structure: (**A**) assessment of visual acuity over time demonstrates a significant decrease in both untreated EAE mice and GA-treated EAE animals as of 21 days after EAE induction. More importantly, EAE mice in the GA treatment cohort show a solid preservation in visual acuity thereafter with a trend towards recovery. Mean data are given with SEM, **** *p* < 0.0001 in EAE vs. naïve, ^#^ *p* < 0.05, ^##^ *p* < 0.01, ^###^ *p* < 0.001 in EAE vs. EAE + GA, and ^§^ *p* < 0.05 in EAE + GA vs. naïve. (**B**) Analysis of the GCL/IPL complex using OCT indicates a significant decrease in thickness in untreated EAE mice whereas GA treatment significantly ameliorates GCL/IPL complex thinning. (**C**) Cumulative pattern ERG tracing results from each group demonstrate a clear reduction in the amplitudes in untreated EAE mice and a preserved pattern ERG waveform in EAE animals having received GA. (**D**) The P1-N2 amplitude in EAE mice is significantly reduced when compared to GA-treated EAE animals as well as naïve mice. Data shown in (**B**) and (**D**) represent individual eyes with mean ± SD.

The visual acuity levels of all the animals of the second EAE cohort were tested at baseline prior to EAE-ON induction and weekly thereafter. The overall baseline visual acuity in the naïve control mice remained unchanged at an average of 0.39 ± 0.01 c/d in the naïve mice throughout the 56-day time course. The visual acuity of the untreated EAE mice was significantly diminished beginning at week 3 compared to the naïve controls with almost no improvement. The GA-treated EAE mice showed a significant preservation of visual acuity compared to the untreated EAE animals with a notable improvement over the 56 days (c/d at day 56: EAE: 0.23 ± 0.08 c/d vs. EAE + GA: 0.34 ± 0.09 c/d; *p* = 0.0001, Figure 4a).

Since cholesterol is necessary for maintaining normal retinal structure and function, we examined structural changes in the retinas of all the EAE mice, specifically within the GCL/IPL complex, using OCT. As anticipated, the untreated EAE animals had a significant thinning of the GCL/IPL complex compared to the naïve controls (EAE: 59 ± 4 µm vs. naive: 66 ± 4 µm, *p* = 10 × 10^−10^). The GA treatment in the EAE animals significantly lessened GCL/IPL complex thinning compared to the untreated EAE animals (EAE: 59 ± 4 µm vs. EAE + GA: 61 ± 4 µm *p* = 0.023); however, the GCL/IPL complex was still significantly thinner than naïve controls (*p* = 9.5 × 10^−10^, Figure 4b).

Since we found increased synaptogenesis in the GA-supplemented retinal organoids, we utilized pattern ERG to examine the effect of GA on the visual function of EAE mice. The electric activity of RGCs significantly contributes to the pattern ERG signal and a decline in the pattern ERG amplitude usually signifies RGC dysfunction [38]. As expected, the waveforms of the untreated EAE animals showed a depressed P1 peak and a shallow N2 trough (Figure 4c) with a P1 to N2 amplitude that was significantly reduced compared to the naïve controls (EAE: 16 ± 4 µV vs. EAE + GA: 24 ± 4 µV; *p* = 1 × 10^−9^, Figure 4d). The waveform in the GA-treated EAE animals demonstrated an improvement with a significantly higher P1 to N2 amplitude compared to the untreated EAE animals (EAE: 16 ± 4 µV vs. EAE + GA: 20 ± 4 µV; *p* = 0.034, Figure 4d). However, similar to the OCT data, the P1 to N2 amplitude of the GA-treated EAE mice was still significantly reduced compared to the naïve controls (EAE + GA: 20 ± 4 µV vs. naïve: 24 ± 4 µV; *p* = 0.013, Figure 4d).

### 3.5. Gentisic Acid Preserves Myelination, Reduces Inflammation, and Rescues Abca1 Expression in Optic Nerves of EAE Mice

**Figure 5:**
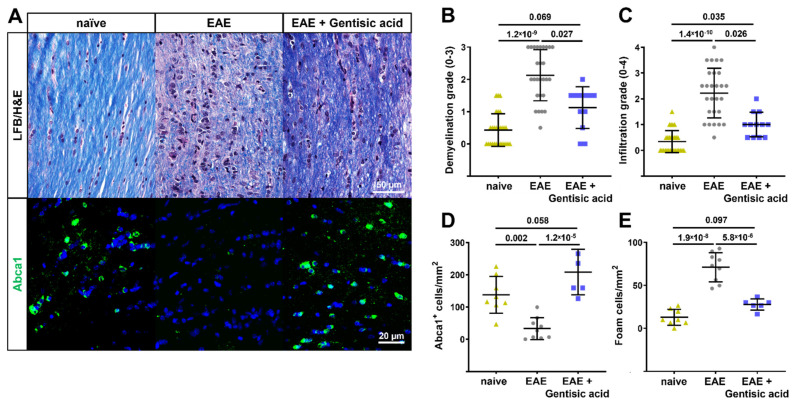
Optic nerve histopathology in EAE mice. (**A**) Representative images of longitudinal optic nerve sections stained with LFB/H&E and Abca1 immunohistochemistry. (**B**) Gentisic acid (GA) treatment of EAE mice preserved the myelin sheath when compared to untreated EAE animals, as indicated by ordinal gradings of demyelination. (**C**) Similarly, EAE mice treated with GA demonstrated reduced cell infiltration whereas untreated EAE mice showed massive grades of infiltration, which is an indication of active optic neuritis. (**D**) Furthermore, systemic GA administration rescued the number of Abca1^+^ cells present and significantly reduced the number of foam cells (**E**) in EAE optic nerves. All data are given as mean ± SD.

The optic nerves were sectioned, stained for myelin using LFB, and graded for demyelination using a 0–3 scale. The optic nerves from the EAE animals demonstrated significant demyelination compared to the naïve controls (EAE: 2.1 ± 0.8 vs. naive: 0.4 ± 0.5; *p* = 1.2 × 10^−9^). The GA treatment of the EAE mice preserved myelin when compared to the untreated EAE animals (EAE + GA: 1.1 ± 0.6; *p* = 0.027) without significant demyelination compared to the naïve controls (Figure 5a,b).

The LFB/H&E-stained optic nerves were graded for cellular infiltration on a 0–4 scale. Greater cellular infiltration was observed in the optic nerves from the untreated EAE animals compared to the naïve controls (EAE: 2.2 ± 1 vs. naive: 0.3 ± 0.4; *p* = 1.4 × 10^−10^) as well as to the EAE mice treated with GA (EAE + GA: 1 ± 0.5; *p* = 0.026). The cellular infiltration of the optic nerves of the GA-treated EAE animals, while reduced compared to the untreated EAE animals, was still significantly higher than the naïve controls (*p* = 0.035, Figure 5c).

To confirm the effects of GA on Abca1 in vivo, we used immunofluorescence to examine the optic nerves for Abca1-positive cells. This was then quantified in representative sections from each animal. The untreated EAE animals had significant reductions in *Abca1*-positive cells compared to the naïve controls (EAE: 33 ± 34 Abca1^+^/mm vs. naïve: 138 ± 57 *Abca*1^+^/mm; *p* = 0.002). As expected, the optic nerves from the GA-treated animals demonstrated significantly increased quantities of Abca1-positive cells (EAE + GA: 208 ± 34 Abca1^+^/mm), which were similar to the naïve controls and significantly higher than the untreated EAE animals (EAE: 33 ± 34 Abca1^+^/mm vs. EAE + GA: 208 ± 34 Abca1^+^/mm; *p* = 1.2 × 10^−5^, Figure 5a,d).

Vice versa, the increase in Abca1-positive cells in the EAE optic nerves after the GA treatment should, logically, decrease the number of foam cells. Foam cells were identified by their unique morphology, featuring lipid laden vacuoles and a foamy appearance in the H&E-stained sections. The optic nerves from the untreated EAE animals contained significantly more foam cells when compared to the GA-treated EAE animals (EAE: 71 ± 17 foam cells/mm vs. EAE + GA: 28 ± 7 foam cells/mm; *p* = 5.8 × 10^−6^) as well as the naïve controls (13 ± 9 foam cells/mm, *p* = 1.9 × 10^−8^). The number of foam cells in the optic nerves of the GA-treated EAE animals was not significantly different from the naïve controls (*p* = 0.097, Figure 5e).

## 4. Discussion

GA is a compound that increases the expression of Abca1, a key efflux transporter for the natural processing of cholesterol in the CNS and retina [21,27]. Our study first aimed to evaluate the effects of GA on cholesterol synthesis, synaptogenesis, and myelination in vitro using human iPSC-derived organoids. We then aimed to discover whether an administration of GA would mitigate the motor-sensory and visual deficits seen in EAE-ON utilizing our MOG_35–55_ EAE model. Our results showed that GA in organ-specific organoids impacts retinal maturation, synapse formation, and myelination. The data also demonstrated that a GA administration in EAE-ON subjects reduces visual and motor-sensory deficits in vivo while reducing inflammation and preserving myelination in the optic nerve. Our results indicate that an improved *Abca1* expression via treatment with GA has pleiotropic effects including the regulation of cholesterol homeostasis required for RGC functioning and the regulation of cholesterol-rich myelin debris to reduce inflammation in the optic nerve.

Cholesterol is essential in the retina for the maintenance of synaptic plasticity [22]. In a previous study, we showed that the dysregulation of cholesterol homeostasis is present in EAE-ON subjects together with a downregulation of *Abca1* that can be rescued by mesenchymal stem cell treatment [20]. Herein, we demonstrated that a GA supplementation to retinal organoids increases the expression of Abca1, as well as enzymes such as HMG-CoA Synthase 1 and Squalene synthase, all key components involved in de novo cholesterol synthesis. The GA treatment improved synapse formation, which was evidenced by the increased expression of *SV2C, GLAST*, and *PSD95* and the accelerated development of neurons including GFP^+^RGCs in the GA-treated retinal organoids. Thus, our data suggest that GA benefits neuronal cholesterol homoeostasis in conjunction with increased synaptic formation and RGC development and thereby accelerates neuronal plasticity. These neuroprotective abilities are effective at mitigating structural GCL/IPL complex degeneration in EAE mice that received systemically administered GA. Furthermore, the GA-treated EAE mice showed improvements in visual acuity and in the pattern ERG amplitude. It is conceivable that this rescue effect may be related to increased neuro-retinal plasticity—as indicated by an increase in PSD95 expression—in EAE mice because the data show that RGC plasticity significantly contributes and correlates with functional and structural changes in optic neuropathies [40].

It was previously known that during demyelination, microglia consume cholesterol-rich myelin debris and export cholesterol to the extracellular space via Abca1 [21]. Demyelination, such as in MS or in the EAE-ON model, produces cholesterol-rich myelin debris that overwhelm the microglia’s ability to recycle cholesterol; thus, these resident brain macrophages develop into pro-inflammatory foamy macrophages. In the GA-treated EAE mice, we observed an increase in Abca1 positive cells in the optic nerve, similar to what was observed in the organoids. The optic nerves from the GA-treated animals also demonstrated reduced inflammation, which is accompanied by a decreased number of foam cells, as well as the preservation of the myelin sheath. Together, this indicates that GA treatment increases *Abca1* and the microglia’s ability to recycle cholesterol, which benefits the recovery of vision and mitigates structural damage in white matter tracts.

These two pathways by which GA and Abca1 impact the visual system are distinct but uniquely intertwined through neuronal activity. Electrically active demyelinated axons have a higher likelihood of inducing remyelination [41]. Therefore, improved synaptic plasticity, as was seen in the retinal organoids, should increase myelination by increasing neuronal activity. Altogether, our data indicate that a GA treatment provides neuroprotection for the retina and optic nerve in MS-like ON via improved cholesterol homeostasis and transport.

Our study demonstrated a proof of concept, as the GA administration reduced disease progression with an increase in Abca1. However, our study has some limitations. For example, we only examined one dose of GA. Secondly, our treatment was prophylactic, started at the day of EAE induction. While this is acceptable as a proof-of-concept study, it is less than ideal for predicting the compound’s efficacy in an interventional treatment scenario. Our results are also consistent with the major mechanism of the drugs modulating anti-inflammation in, for example, rheumatic fever [42], most likely via the inhibition of cyclooxygenase [29], and in macrophage-driven obesity-related chronic inflammation [43]. These mechanisms are likely to further contribute to improved visual and motor-sensory outcomes in GA-treated EAE mice. Due to these limitations, future studies are needed to examine dose dependencies and should include both interventional and late treatment regimens after the disease’s onset, as well as a withdrawal setting. This setup would allow us to mimic the clinical presentation of MS-related ON more closely and would increase translational rigor. To account for GA’s multidimensional effects, future studies should also implement approaches for selective *Abca1* deletion or inhibitors to block the effects of GA to further dissect the cell-specific mechanisms of action.

On the other hand, the highly translational readouts we performed are one of the many strengths of this study. The assessments of visual acuity, OCT imaging, and electrophysiology recordings can be directly translated to clinical uses for future clinical trials. All the EAE mice showed motor sensory deficits and histopathological evidence of optic neuritis, thereby proving our EAE method successfully recapitulates a clinical MS scenario. Our statistics show significant differences between GA-treated and untreated EAE cohorts, attesting to the good analytic power afforded by the group size that accounted for animal dropouts and missing data.

What do the findings of this study mean for those affected? MS, ON, and the associated lasting visual deficits present immense socioeconomic burdens on patients, families, and the healthcare system. Current therapies lack the potential to reverse the progression of vision loss or motor deficits; thus, there is a rationale to develop therapies that can repair myelin damage and protect neurons from further loss and to ultimately reverse symptoms [44]. Herein, we present GA as an inexpensive compound with the ability to improve cholesterol recycling, which could prove to be a potential therapy for rehabilitation after demyelinating disease of the optic nerve.

## Figures and Tables

**Table 1:** PCR Targets and Sequences.

Target	Forward Sequence	Reverse Sequence
*ABCA1*	5′-GCTACCCACCCTATGAACAAC-3′	5′-AGATAATCCCCTGAACCCAAG-3′
*SV2C*	5′-GCTCTGCATGTTCTGGATGA-3′	5′-GATGACAAACACACGCCAAC-3′
*PSD95 (DLG4)*	5′-ATACCGCTACCAAGATGAAGAC-3′	5′-TCACCTGCAACTCATATCCTG-3′
*GLAST (SLC1A3)*	5′-GAGGATGTTACAGATGCTGGTC-3′	5′-TAATAGACTACAGCTCGCATTCC-3′
*HMGCS1*	5′GAAACAGTGACAGACCTGGAG-3′	5′-AGCAAGCTTCTGCATTCAAAG-3′
*TUJ1 (TUBB3)*	5′-GGCCTTTGGACATCTCTTCAG-3′	5′-CCTCCGTGTAGTTGACCCTT-3′
*SQS (FDFT1)*	5′-ACAACCTGGTGCGCTTC-3′	5′-GATAACAGCTGCGAAACTGC-3′
*TBP*	5′-GCTGTTTAACTTCGCTTCCG-3′	5′-CAGCAACTTCCTCAATTCCTTG-3′
*MBP*	5′-GGCCGGACCCAAGATGAAAA-3′	5′-CCCCAGCTAAATCTGCTCAGG-3′
*MYRF*	5′-CAGTCCCAGTCAGACCGGA-3′	5′-CCCTTCTTACGCATGTTGTTAGC-3′
*PLP1*	5′-ACCTATGCCCTGACCGTTG-3′	5′-TGCTGGGGAAGGCAATAGACT-3′
*PDGFR*	5′-TTGAAGGCAGGCACATTTACA-3′	5′-GCGACAAGGTATAATGGCAGAAT-3′
*OLIG2*	5′-TGGCTTCAAGTCATCCTCGTC-3′	5′-ATGGCGATGTTGAGGTCGTG-3′
*NG2 (CSPG4)*	5′-AGGACGAAGGAACCCTAGAGT-3′	5′-CACAGGCACACTGTTGTGGA-3′

## Data Availability

Data will be made available upon request. Please contact the corresponding author.

## References

[1] Bennett J.L. (2019). Optic Neuritis. Contin. (Minneapolis, Minn.).

[2] Shams P.N., Plant G.T. (2009). Optic neuritis: A review. Int. MS J..

[3] Beck R.W., Cleary P.A., Anderson M.M., Keltner J.L., Shults W.T., Kaufman D.I., Buckley E.G., Corbett J.J., Kupersmith M.J., Miller N.R. (1992). A randomized, controlled trial of corticosteroids in the treatment of acute optic neuritis. The Optic Neuritis Study Group. N. Engl. J. Med..

[4] Beck R.W., Gal R.L., Bhatti M.T., Brodsky M.C., Buckley E.G., Chrousos G.A., Corbett J., Eggenberger E., Goodwin J.A., Katz B. (2004). Visual function more than 10 years after optic neuritis: Experience of the optic neuritis treatment trial. Am. J. Ophthalmol..

[5] De Lott L.B., Bennett J.L., Costello F. (2022). The changing landscape of optic neuritis: A narrative review. J. Neurol..

[6] Sabadia S.B., Nolan R.C., Galetta K.M., Narayana K.M., Wilson J.A., Calabresi P.A., Frohman E.M., Galetta S.L., Balcer L.J. (2016). 20/40 or Better Visual Acuity After Optic Neuritis: Not as Good as We Once Thought?. J. Neuroophthalmol..

[7] Petzold A., Balcer L.J., Calabresi P.A., Costello F., Frohman T.C., Frohman E.M., Martinez-Lapiscina E.H., Green A.J., Kardon R., Outteryck O. (2017). Retinal layer segmentation in multiple sclerosis: A systematic review and meta-analysis. Lancet Neurol..

[8] Vabanesi M., Pisa M., Guerrieri S., Moiola L., Radaelli M., Medaglini S., Martinelli V., Comi G., Leocani L. (2019). In vivo structural and functional assessment of optic nerve damage in neuromyelitis optica spectrum disorders and multiple sclerosis. Sci. Rep..

[9] Arneth B. (2021). Contributions of T cells in multiple sclerosis: What do we currently know?. J. Neurol..

[10] Bar-Or A., Li R. (2021). Cellular immunology of relapsing multiple sclerosis: Interactions, checks, and balances. Lancet Neurol..

[11] Murray T.J. (2006). Diagnosis and treatment of multiple sclerosis. BMJ.

[12] Derfuss T., Mehling M., Papadopoulou A., Bar-Or A., Cohen J.A., Kappos L. (2020). Advances in oral immunomodulating therapies in relapsing multiple sclerosis. Lancet Neurol..

[13] Fogarty E., Schmitz S., Tubridy N., Walsh C., Barry M. (2016). Comparative efficacy of disease-modifying therapies for patients with relapsing remitting multiple sclerosis: Systematic review and network meta-analysis. Mult. Scler. Relat. Disord..

[14] Tselis A., Perumal J., Caon C., Hreha S., Ching W., Din M., Van Stavern G., Khan O. (2008). Treatment of corticosteroid refractory optic neuritis in multiple sclerosis patients with intravenous immunoglobulin. Eur. J. Neurol..

[15] Ruprecht K., Klinker E., Dintelmann T., Rieckmann P., Gold R. (2004). Plasma exchange for severe optic neuritis: Treatment of 10 patients. Neurology.

[16] Gugliandolo A., Bramanti P., Mazzon E. (2020). Mesenchymal Stem Cells in Multiple Sclerosis: Recent Evidence from Pre-Clinical to Clinical Studies. Int. J. Mol. Sci..

[17] Florou D., Katsara M., Feehan J., Dardiotis E., Apostolopoulos V. (2020). Anti-CD20 Agents for Multiple Sclerosis: Spotlight on Ocrelizumab and Ofatumumab. Brain Sci..

[18] Lavrnja I., Smiljanic K., Savic D., Mladenovic-Djordjevic A., Tesovic K., Kanazir S., Pekovic S. (2017). Expression profiles of cholesterol metabolism-related genes are altered during development of experimental autoimmune encephalomyelitis in the rat spinal cord. Sci. Rep..

[19] Berghoff S.A., Spieth L., Sun T., Hosang L., Schlaphoff L., Depp C., Duking T., Winchenbach J., Neuber J., Ewers D. (2021). Microglia facilitate repair of demyelinated lesions via post-squalene sterol synthesis. Nat. Neurosci..

[20] Gramlich O.W., Brown A.J., Godwin C.R., Chimenti M.S., Boland L.K., Ankrum J.A., Kardon R.H. (2020). Systemic Mesenchymal Stem Cell Treatment Mitigates Structural and Functional Retinal Ganglion Cell Degeneration in a Mouse Model of Multiple Sclerosis. Transl. Vis. Sci. Technol..

[21] Cantuti-Castelvetri L., Fitzner D., Bosch-Queralt M., Weil M.T., Su M., Sen P., Ruhwedel T., Mitkovski M., Trendelenburg G., Lutjohann D. (2018). Defective cholesterol clearance limits remyelination in the aged central nervous system. Science.

[22] Hussain G., Wang J., Rasul A., Anwar H., Imran A., Qasim M., Zafar S., Kamran S.K.S., Razzaq A., Aziz N. (2019). Role of cholesterol and sphingolipids in brain development and neurological diseases. Lipids Health Dis..

[23] Pikuleva I.A., Curcio C.A. (2014). Cholesterol in the retina: The best is yet to come. Prog. Retin. Eye Res..

[24] Sene A., Khan A.A., Cox D., Nakamura R.E., Santeford A., Kim B.M., Sidhu R., Onken M.D., Harbour J.W., Hagbi-Levi S. (2013). Impaired cholesterol efflux in senescent macrophages promotes age-related macular degeneration. Cell Metab..

[25] Ban N., Lee T.J., Sene A., Choudhary M., Lekwuwa M., Dong Z., Santeford A., Lin J.B., Malek G., Ory D.S. (2018). Impaired monocyte cholesterol clearance initiates age-related retinal degeneration and vision loss. JCI Insight.

[26] Storti F., Klee K., Todorova V., Steiner R., Othman A., van der Velde-Visser S., Samardzija M., Meneau I., Barben M., Karademir D. (2019). Impaired ABCA1/ABCG1-mediated lipid efflux in the mouse retinal pigment epithelium (RPE) leads to retinal degeneration. Elife.

[27] Ananth S., Gnana-Prakasam J.P., Bhutia Y.D., Veeranan-Karmegam R., Martin P.M., Smith S.B., Ganapathy V. (2014). Regulation of the cholesterol efflux transporters ABCA1 and ABCG1 in retina in hemochromatosis and by the endogenous siderophore 2,5-dihydroxybenzoic acid. Biochim. Biophys. Acta.

[28] Abedi F., Razavi B.M., Hosseinzadeh H. (2020). A review on gentisic acid as a plant derived phenolic acid and metabolite of aspirin: Comprehensive pharmacology, toxicology, and some pharmaceutical aspects. Phytother. Res..

[29] Holmes T.J., Vennerstrom J.L., John V. (1985). Inhibition of cyclooxygenase mediated by electrochemical oxidation of gentisic acid. J. Biol. Chem..

[30] Nakano T., Ando S., Takata N., Kawada M., Muguruma K., Sekiguchi K., Saito K., Yonemura S., Eiraku M., Sasai Y. (2012). Self-formation of optic cups and storable stratified neural retina from human ESCs. Cell Stem Cell.

[31] Cheng L., Cring M.R., Wadkins D.A., Kuehn M.H. (2022). Absence of Connexin 43 results in smaller retinas and arrested, depolarized retinal progenitor cells in human retinal organoids. Stem Cells.

[32] Madhavan M., Nevin Z.S., Shick H.E., Garrison E., Clarkson-Paredes C., Karl M., Clayton B.L.L., Factor D.C., Allan K.C., Barbar L. (2018). Induction of myelinating oligodendrocytes in human cortical spheroids. Nat. Methods.

[33] Schmittgen T.D., Livak K.J. (2008). Analyzing real-time PCR data by the comparative C(T) method. Nat. Protoc..

[34] Nafees S., Ahmad S.T., Arjumand W., Rashid S., Ali N., Sultana S. (2012). Modulatory effects of gentisic acid against genotoxicity and hepatotoxicity induced by cyclophosphamide in Swiss albino mice. J. Pharm. Pharm..

[35] Bittner S., Afzali A.M., Wiendl H., Meuth S.G. (2014). Myelin oligodendrocyte glycoprotein (MOG35-55) induced experimental autoimmune encephalomyelitis (EAE) in C57BL/6 mice. J. Vis. Exp. JoVE.

[36] Prusky G.T., Alam N.M., Beekman S., Douglas R.M. (2004). Rapid quantification of adult and developing mouse spatial vision using a virtual optomotor system. Investig. Ophthalmol. Vis. Sci..

[37] Zeng H., Dumitrescu A.V., Wadkins D., Elwood B.W., Gramlich O.W., Kuehn M.H. (2022). Systemic Treatment with Pioglitazone Reverses Vision Loss in Preclinical Glaucoma Models. Biomolecules.

[38] Chou T.H., Bohorquez J., Toft-Nielsen J., Ozdamar O., Porciatti V. (2014). Robust mouse pattern electroretinograms derived simultaneously from each eye using a common snout electrode. Investig. Ophthalmol. Vis. Sci..

[39] Schwabenland M., Bruck W., Priller J., Stadelmann C., Lassmann H., Prinz M. (2021). Analyzing microglial phenotypes across neuropathologies: A practical guide. Acta Neuropathol..

[40] Porciatti V., Ventura L.M. (2012). Retinal ganglion cell functional plasticity and optic neuropathy: A comprehensive model. J. Neuroophthalmol..

[41] Gautier H.O., Evans K.A., Volbracht K., James R., Sitnikov S., Lundgaard I., James F., Lao-Peregrin C., Reynolds R., Franklin R.J. (2015). Neuronal activity regulates remyelination via glutamate signalling to oligodendrocyte progenitors. Nat. Commun..

[42] Smith M.J., Pharm M. (1952). Some new drugs in the treatment of rheumatic fever. Postgrad. Med. J..

[43] Kang M.J., Choi W., Yoo S.H., Nam S.W., Shin P.G., Kim K.K., Kim G.D. (2021). Modulation of Inflammatory Pathways and Adipogenesis by the Action of Gentisic Acid in RAW 264.7 and 3T3-L1 Cell Lines. J. Microbiol. Biotechnol..

[44] Bove R.M., Green A.J. (2017). Remyelinating Pharmacotherapies in Multiple Sclerosis. Neurotherapeutics.

